# Unconventional T cells in anti-cancer immunity

**DOI:** 10.3389/fimmu.2025.1618393

**Published:** 2025-07-17

**Authors:** Ariel Laub, Nathalia Rodrigues de Almeida, Shouxiong Huang

**Affiliations:** ^1^ Host-Pathogen Interactions Program, Texas Biomedical Research Program, San Antonio, TX, United States; ^2^ Department of Molecular Microbiology and Immunology, University of Texas at San Antonio, San Antonio, TX, United States

**Keywords:** unconventional T cells, polar metabolites, lipids, cancer, immunotherapy, MHC class I-related protein 1 (MR1), CD1

## Abstract

Unlike conventional T cells that detect peptide antigens loaded to major histocompatibility complex (MHC) molecules, unconventional T cells respond to non-peptidic metabolite antigens presented by MHC class I-like proteins, such as CD1 and MHC-related protein 1 (MR1). Semi-invariant mucosal-associated invariant T (MAIT) cells, γδ T cells, and invariant natural killer T (iNKT) cells, together with other CD1- or MR1-restricted T cell subsets expressing diverse T cell receptors (TCR), elicit an innate-like response independent of diverse MHC genetics. In contrast to an overall enhanced response to bacterial-derived riboflavin precursor metabolites in infections, MAIT cells often exhibit an immunosuppressive or exhausted phenotype in glioblastoma, lung cancer, colorectal cancer, and various hematological malignancies. Whereas some tumor cells can activate MAIT cells, the structures and functions of tumor-derived MR1 ligands remain largely unknown. Novel discoveries of mammalian-derived agonists and antagonists binding to MR1 protein are our knowledge of MR1 ligand structures and functions from MAIT cell activation in healthy conditions to anti-cancer immunity. Recent findings reveal that nucleoside and nucleobase analogs, as self-metabolites to activate MR1-restricted T cells, are regulated in the tumor microenvironment. Likewise, iNKT cells exhibit a dynamic role in cancer, capable of both protumor and antitumor immunity. Similarly, γδ T cells have also demonstrated both protective and tumor-promoting roles, via recognizing stress-induced protein and metabolite ligands. This review further depicts the distinct kinetics of responses, highlighting a rapid activation of unconventional T cells in solid versus hematological cancers. Emerging therapeutic strategies, including antigen-loaded MR1 and CD1, adoptive T cell transfer, chimeric antigen receptor-T (CAR-T) cells, T cell receptor-T (TCR-T) cells, and combination treatments with immune checkpoint inhibitors, yet remain challenging, hold promise in overcoming tumor-induced immunosuppression and genetic restriction of conventional T cell therapies. By addressing critical gaps, such as novel structures and functions of cancer metabolite antigens, unconventional T cells offer unique advantages in anti-cancer immunotherapy.

## Introduction

Antitumor T cell immunity against malignancy has been generally focused on studying conventional T cell activation, which relies on recognizing tumor peptide antigens presented by polymorphic major histocompatibility complex (MHC) or human leukocyte antigen (HLA) class I and II molecules in various human populations (1, 2). Conventional cytotoxic CD8^+^ T lymphocytes (CTLs) recognize peptide antigens presented by MHC class I, and CD4^+^ T cells engage with peptide-MHC class II complexes (3), driving crucial anticancer immune responses and framing cancer immunotherapies (4, 5). These adaptive T cells, particularly CD8^+^ CTLs, mediate tumor cell killing through antigen-specific recognition of tumor-associated antigens (TAAs) or neoantigens, eliciting potent cytotoxic molecular mediators capable of direct tumor lysis. However, tumor cells often evade this response by downregulating MHC class I expression or inducing an immunosuppressive tumor microenvironment (TME), limiting the effectiveness of conventional T cells and leading to immune escape for cancer progression (5). In contrast, unconventional T cells rely on recognizing polar or lipid metabolite antigens presented by non-classical MHC class I or MHC class Ib molecules with limited polymorphisms. These include lipids by the Cluster of Differentiation 1 (CD1) proteins for CD1-restricted T cells and polar metabolites by MHC-related protein 1 (MR1) for MR1-restricted T cells (6–13). Notably, the non-classical antigen presentation mechanisms allow unconventional T cells to bypass MHC restriction, enabling rapid and individual-unrestricted immune activation that does not rely on genetically diverse classical HLA proteins in various human populations (6, 12, 14).

Unconventional T cells are generally first classified based on antigen presentation mechanisms for activation. CD1-restricted T cells can recognize bacterial and mammalian lipids to exert various immune regulation and effector responses (10, 15). Particularly, CD1d-restricted invariant natural killer T (iNKT) cells are well-characterized to robustly produce cytokines that enhance both pro-inflammatory and regulatory immune pathways (7). MR1-restricted T cells recognize polar metabolites with bacterial sources mostly from vitamin B biosynthetic pathways, particularly microbial riboflavin precursors (16, 17), and with currently known mammalian ligands mostly from nucleoside metabolism (18–20). γδ T cells are known to detect phosphoantigens through members of the butyrophilin (BTN) family, which form a receptor complex to engage γδ TCR for activation. The γδ T cells also detect CD1-presented lipids and MR1-presented polar metabolites. The capacity to sense cellular stress and metabolite compounds enables unconventional T cells to play key roles in both microbial defense and tumor surveillance (21, 22). Further classification of CD1- or MR1-restricted T cell subsets usually relies on the invariant or diverse TCR sequences. CD1d-restricted T cells are typically divided into invariant NKT cells (iNKT or type I NKT cells) expressing an invariant TCRα chain (human TRAV10 or mouse TRAV11) and diverse NKT cells (dNKT or type II NKT cells) expressing variable TCRα chains (15, 23). Similar to NKT cells, MR1-restricted T cells (MR1T) can be divided into mucosal-associated invariant T (MAIT) cells expressing invariant TCRα chains (human TRAV1-2 or mouse TRAV1) (24) and diverse MR1-restricted T cells expressing diverse TCRs (diverse MR1T, dMR1T, TRAV1-2^-^ MR1T, or Vα7.2^-^ MR1T in humans). The γδ T cells are typically classified into three primary subsets based on their δ chain usage: Vδ1^+^, Vδ2^+^, and Vδ3^+^ (22, 25). This semi-invariant TCR expression and interaction with metabolite antigens presented by limited polymorphic MHC class I-like proteins overall define the innate-like nature of unconventional T cell responses.

In cancer immunity, unconventional T cells generally play dual roles, either promoting tumor clearance through cytotoxicity and cytokine production or, conversely, contributing to tumor progression when exposed to chronic immunosuppressive signaling in the TME and leading to undesirable exhausted phenotypes (26). This review will focus on MR1-restricted MAIT cells and diverse MR1T cells in cancer immunity, comparing their roles to other unconventional T cell subsets, including iNKT cells and γδ T cells. By analyzing their dual nature of immune responses in different tumor settings, we center on the structures and functions of metabolite antigens for unconventional T cell activation in the cancer context and discuss their potential as targets for cancer immunotherapy. Additionally, we highlight emerging therapeutic strategies, primarily by activating semi-invariant and diverse T cells through MR1- and CD1-mediated antigen presentation, harnessing these T cells for adoptive T cell transfer, and combining multiple immune therapies such as checkpoint blockade. Given their ability to bypass MHC restrictions and their innate-like rapid responses, MAIT cells and other unconventional T cells are expected to provide promising opportunities for improving immune-based cancer treatments.

## Self-metabolite antigen presentation

**Figure 1:**
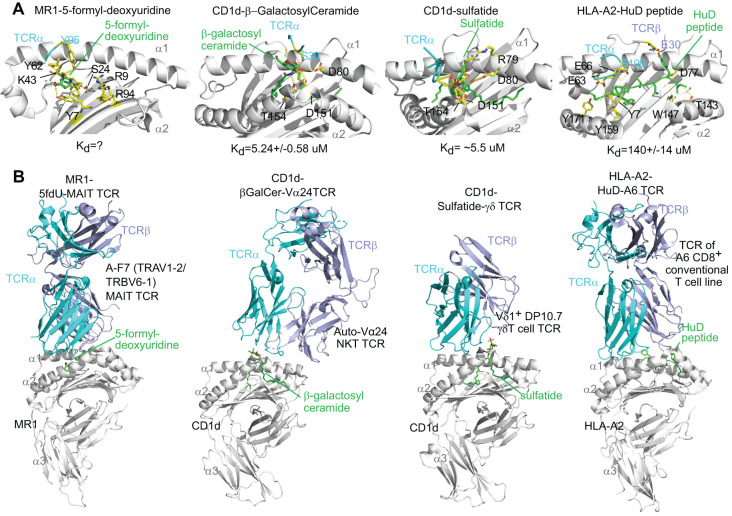
Re-analyses of tertiary crystal structures of MR1 or CD1, mammalian metabolite antigens, and invariant T cell receptors, in parallel with an HLA-A2-auto peptide-TCR complex. The reported crystal structures for human MR1, 5-formyl-deoxyuridine, and A-F7 MAIT cell TCR (9EK7 from the PDB database), human CD1d, β-galactosylceramide, and autoreactive Vα24 TCR (3SDX), human CD1d, sulfatide, and DP10.7 γδ TCR (4MNG), and HLA-A2, glioblastoma peptide HuD, and A6 conventional CD8+ T cell TCR (3PWP) were compared via Pymol for metabolite antigen interaction with MR1, CD1, and TCR chains. **(A)** Metabolite antigen binding to MR1, CD1, and TCR chains is shown through polar interactions (yellow dots), particularly hydrogen bonding, but hydrophobic interaction is not shown. Interacting residues were annotated. α1, α2, and α3 label α1, α2, and α3 domains with α2 domains partially removed to show protein-ligand interactions. Kd, the dissociation constant, represents the concentration of ligand binding to 50% of receptor molecules. **(B)** Metabolite antigen binding to MHC class I-like proteins differentially shapes the orientation of TCRα and TCRβ chains.

Binary and tertiary structures of antigen-presentation complex containing an MHC class I-like protein and a self-metabolite antigen with or without a TCR provide critical knowledge on the topology, interacting sites, and affinity binding of metabolite antigens with proteins. Different from peptide antigens that generally use multiple hydrogen bonds to interact with classical MHC class I proteins, metabolite antigens bind a smaller number of amino acid residues via polar and hydrophobic interactions, and even covalent bonds to interact with CD1 and MR1 proteins (**Figure 1A**) (27–30). Specifically, polar metabolite antigens such as nucleoside derivatives or riboflavin intermediate metabolites are small in size and reasonably interact with a small number of MR1 residues. Some mammalian cell-derived polar metabolites, such as the carbonyl adduct of adenine (18) and 5-formyl-deoxyuridine (30), form a Schiff’s base with lysine 43 (K43) of human MR1, similarly to the bacterial riboflavin precursor metabolites 5-[2-oxopropylideneamino]-6-D-ribitylaminouracil (5-OP-RU) (16). Lipid metabolites generally consist of hydrophilic head groups to form polar interactions with CD1 proteins and TCR chains, while hydrophobic fatty acyl or sphingosine chains interact with the ligand-binding clefts of CD1 proteins through hydrophobic interactions (**Figure 1A**) (29). Binding affinity of mammalian cell metabolite antigens to MR1 or CD1 proteins is detected by K_d_, a dissociation constant reflecting the concentration of ligand binding to 50% of receptor molecules, generally at a micromolar range for self-metabolite antigens (**Figure 1A**), different from an overall nanomolar range of K_d_ for non-self-metabolite antigens such as human CD1d binding to marine sponge-derived α-galactosylceramide (α-GalCer) (31) and human MR1 binding to a bacterial metabolite derivative 5-OP-RU (32). By examining the polar interactions between metabolite ligand and invariant TCR, it appears that invariant TCRα chains form major contacts, while TCRβ chains point to different positions away from the center of metabolite antigens, unlike conventional TCRα and TCRβ chains generally center on α1 and α2 domains of the HLA-A2 protein (**Figures 1A, B**) and other classical MHC class I proteins (33). A comparison of these tertiary antigen-presentation complexes depicts an unconventional interacting pattern utilizing less polar interactions and uncentered engagement with TCR at a low affinity for unconventional T cell activation in cancers. Various identified mammalian-derived metabolite compounds for unconventional T cell activation are detailed in the following sections.

## Polar metabolites as MR1 ligands

**Figure 2:**
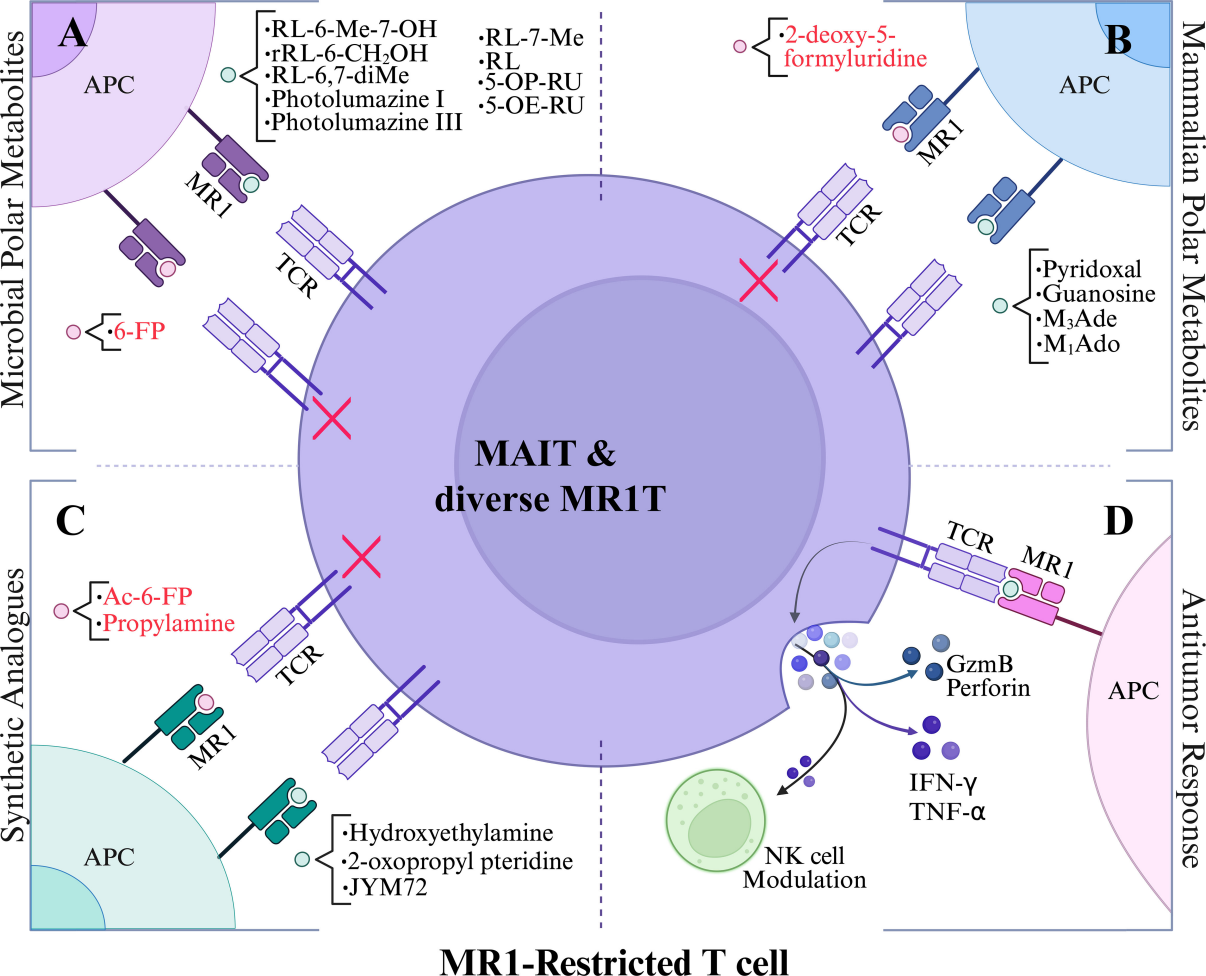
Polar metabolite antigen presentation to MR1-restricted T cells. **(A)** Vitamin B1, B6, and B9 precursors as MR1 ligands. **(B)** tumor-associated mammalian-derived metabolites as MR1 ligands. **(C)** synthetic analogues as MR1 ligands. **(D)** reported antitumor responses upon stimulation with polar metabolites. Black lettering denotes agonists and red lettering depicts antagonists. Red X indicates inhibited MR1T cell response. Abbreviations are as follows: 5-(2-oxopropylideneamino)-6-D-ribitylaminouracil (5-OP-RU), 5-(2-oxoethylideneamino)-6-D-ribitylaminouracil (5-OE-RU), ribityl lumazine (RL), 7-methyl-ribityllumazine (RL-7-Me), 6,7-dimethyl-8-ribityllumazine (RL-6,7-diMe), 6-methyl-7-hydroxy-ribityllumazine (RL-6-Me-7-OH), 3-(2-deoxy-β-D-erythro-pentofuranosyl)-6-(hydroxymethyl)-8-oxo-9H-purine-2-carbaldehyde (M1Ado), 6-(hydroxymethyl)-8-oxo-9H-purine-2-carbaldehyde (M3Ade), and acetyl-6-formylpterin (Ac-6-FP).

MR1 is an MHC class I-like antigen-presentation molecule for presenting polar metabolite antigens to MR1-restricted T cells (6, 34–36). Although surface expression is generally low, MR1 expresses broadly across tissues at RNA and protein levels (11, 37), and activates MAIT cells upon bacterial (38) or cancer metabolite stimulation (20, 39). Similar to classical MHC molecules, MR1 remains largely retained intracellular until it binds a ligand, at which point it is transported to the cell surface for antigen presentation (11, 35–37). This ligand-regulated expression mechanism allows MR1 to serve as a metabolic checkpoint, particularly in recognizing small molecule metabolites derived from microbial riboflavin (16) or mammalian metabolite biosynthesis (18, 20) (**Figure 2**). It was well known that the semi-invariant conserved MAIT TCR predominantly utilizes TRAV1-2 (Vα7.2 in humans) conjuncted with TRAJ33 (Jα33) (40), or with TRAJ12 or TRAJ20 (41) for TCRα chains and a limitedly diverse TCRβ chains (TRBV6 or Vβ13.2~13.5, Vβ6.5~6.8, TRBV20 or Vβ2.1, 2.3) in humans (40). In mice, MAIT cells express Vα19-Jα33 TCRα, mainly paired with Vβ8 and Vβ6 segments (11, 12, 34, 35). Diverse MR1T cells, which do not express TRAV1-2 segment (TRAV1-2^-^) but respond to various metabolites in infection and cancer, were also reported recently (**Figure 2**) (13, 16–19, 32, 42–44).

### Polar microbial metabolites

**Figure 3:**
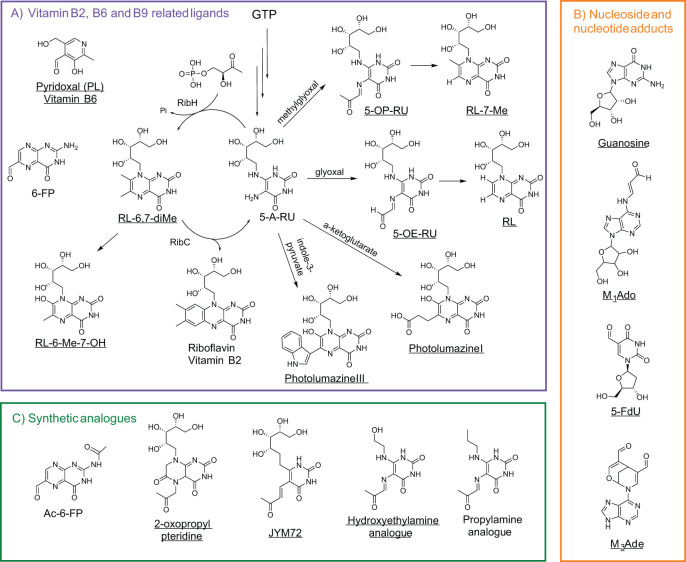
Polar metabolites as MR1 ligands. **(A)** Vitamin B1, B6, and B9 precursors or related metabolites shown in relation to their metabolic pathways (GTP for riboflavin biosynthesis), with reactants labeled above arrows indicating product formation. RibH (lumazine synthase) and RibC (riboflavin synthase) enzymatically convert early vitamin B2 precursors into riboflavin derivatives. **(B)** Nucleoside and nucleoside adducts but with unknown MR1-binding capacity for guanosine and 2-deoxy-5-formyluridine. **(C)** Synthetic analogues associated with vitamin precursor metabolites. Ac-6-FP is derived from vitamin B9 precursors, while 2-oxopropyl pteridine, JYM72 (chemical name unknown), hydroxyethylamine, and propylamine are linked to vitamin B2 derivatives. MR1 agonists or MAIT stimulators are underlined. Abbreviations are as follows: guanosine triphosphate (GTP), 5-amino-6-(D-ribitylamino)uracil (5-A-RU), 5-(2-oxopropylideneamino)-6-D-ribitylaminouracil (5-OP-RU), 5-(2-oxoethylideneamino)-6-D-ribitylaminouracil (5-OE-RU), ribityl lumazine (RL), 6,7-dimethyl-8-ribityllumazine (RL-6,7-diMe), 6-methyl-7-hydroxy-ribityllumazine (RL-6-Me-7-OH), pyridoxal (PL), 2-deoxy-5-formyluridine (fdU), 3-(2-deoxy-β-D-erythro-pentofuranosyl)-6-(hydroxymethyl)-8-oxo-9H-purine-2-carbaldehyde (M1Ado), 6-(hydroxymethyl)-8-oxo-9H-purine-2-carbaldehyde (M3Ade), and acetyl-6-formylpterin (Ac-6-FP).

Most identified MAIT cell antigens may not be directly associated with cancer but have been used for MAIT cell activation in cancer cell killing assays. The first identified MR1 ligand, 6-formylpterin (6-FP), was reported in 2012 from the refolded MR1 protein potentially binding with nutrient metabolites from culture media, and it was a photodegradation product of folic acid (vitamin B9) (17). 6-FP and its synthetic analog acetyl-6-formylpterin (Ac-6-FP) bind to MR1 and potently upregulate the cell surface expression of MR1 on human lymphoid C1R cells, but generally inhibit MAIT cells activation (17, 41). Both 6-FP and Ac-6-FP function as competitive inhibitors and stain MAIT or diverse MR1T cells, indicating possible roles in immune modulation rather than robust effector function. Further, MAIT cells are known to recognize derivatives of the bacterial riboflavin (Vitamin B2) intermediate 5-Amino-6-(D-ribitylamino) uracil (5-A-RU), such as 5-(2-oxoethylideneamino)-6-D-ribitylaminouracil (5-OE-RU) and 5-[2-oxopropylideneamino]-6-D-ribitylaminouracil (5-OP-RU), as potent MAIT cell activators, formed by the non-enzymatic condensation of 5-A-RU with small carbonyl metabolites such as glyoxal and methylglyoxal, respectively (16, 41) (**Table 1**; **Figure 3**). Among these, 5-OP-RU remains the most potent activator of human and mouse MAIT cells. It induces robust cytokine production and has demonstrated pronounced antitumor activity in murine models of liver, lung, and subcutaneous tumors. Notably, pre-pulsing B16F10 melanoma cells with 5-OP-RU enhances MAIT cell-mediated tumor control, partly through modulation of NK cell responses (45, 46), although, in a different context, MR1-expressing B16F10 cells can suppress NK cell frequency via MAIT activation (47). In contrast, 5-OE-RU is formed with a condensation reaction with glyoxal rather than methylglyoxal and also activates MAIT TCR-expressing Jurkat cells, but with reduced potency (16).

**Table 1:** Mammalian and bacterial polar metabolites regulating MAIT cell responses.

MR1 ligands	Detection in cancer or bacterial cells	*In vitro* model	T cell activation	Effector molecules	Anti-cancer T cell immunity	References
5-(2-oxopropylideneamino) -6-d-ribitylaminouracil (5-OP-RU)	Mass spectrometry (MS) detection of *m/z* 329.11 ([M-H]^-^) from recombinant MR1 refolded with the culture supernatant of *E. coli* (DH5α), *Lactococcus lactis* (CB013), or products of 5-A-RU and methylglyoxal.	*(i)* CT26 colon cancer cells, RIL-175 HCC cells engrafted in C57BL/6 mice. *(ii)*5-OP-RU-pulsed B16F10 melanoma cells	*(i)* ↑CD69; MAIT cells activated by 5-OP-RU and CpG	*(i)* IFN-γ, Perforin, Grzm B; ↓IL-17A	*(i)* Activated MAIT cells show pronounced and consistent antitumor activity and a prolonged survival of liver tumors, lung metastases, and a subcutaneous tumor in a mouse model. *(ii)* MAIT cell activation and expansion with 5-OP-RU or 5-OP-RU-pulsed B16F10 melanoma cells enhances antitumor immunity by modulation of NK cell activity and increases cancer cell lysis.	(16, 45, 46)
5-(2-oxoethylideneamino)-6-dribitylaminouracil (5-OE-RU)	MS detection of *m/z* 315.09 ([M-H]^-^) from recombinant MR1 refolded with the culture supernatant of *E. coli* (DH5α), or *S. typhimurium*, or the products of 5-A-RU and glyoxal.	Jurkat.MAIT TCR overexpressed cells	↑CD69	–	MAIT cell activation to a lesser extent compared to 5-OP-RU.	(16)
reduced 6-hydroxymethyl-8-Dribityllumazine (rRL-6-CH_2_OH)	Fragmentation of synthetic rRL-6-CH_2_OH with a parental ion *m/z* 315.09 ([M-H]^-^) matches its predicted fragments.	Jurkat.MAIT cells; SKW-MAIT-TRBV6-1 cells	↑CD69	↑IFN-γ, TNF-α	Activation of Jurkat cells with MAIT TCR overexpression and SKW-MAIT-TRBV6-1 cells	(17, 48)
6-methyl-7-hydroxy-8-d-ribityllumazine (RL-6-Me-7-OH)	Predicted from the bacterial riboflavin metabolic pathway with a predicted parental ion at *m/z* 329.11 ([M-H]^-^). Fragments of small molecules from recombinant MR1 expressed in insect cells with *M. smegmatis* or *E.coli* infection match the fragmentation pattern of synthetic compounds with unclear accuracy.	Jurkat.MAIT TRAV1-2-TRAJ33 and TRBV6-1, TRBV6-4, or TRBV20 cells	↑CD69	↑IFN-γ, TNF-α	Activation of Jurkat cells with MAIT TCR overexpression	(17, 43)
6,7-dimethyl-8-d-ribityllumazine (RL-6,7-diMe)	Predicted from bacterial riboflavin metabolic pathway with a predicted parental ion at *m/z* 329.11 ([M-H]^-^)	Jurkat.MAIT TCR overexpressed cells	↑CD69	↑ IFN-γ, TNF-α	Activation of Jurkat cells with MAIT TCR overexpression	(17, 48)
7-methyl-8-ribityllumazine (RL-7-Me)	A product of 5-A-RU condensation with small metabolites was confirmed by nuclear magnetic resonance (NMR), ^1^HNMR (600MHz),^13^C NMR (150MHz), and heteronuclear multiple bond correlation (HMBC, a 2D NMR).	Jurkat.MAIT TRAV1-2-TRBV6-1or TRBV6-4 reporter cells	↑CD69		Weak activation of Jurkat expressing MAIT TCR TRAV1-2–TRBV6-1 (A-F7) and TRAV1-2–TRBV6-4 and poor upregulation of MR1.	(16, 49)
8-d-ribityllumazine (RL)	A product of 5-A-RU condensation with small metabolites was confirmed by NMR. ^1^HNMR (600MHz),^13^C NMR (150MHz) and HMBC.	–	–	–	Undetermined	(16)
6-(1*H*-indol-3-yl)-7-hydroxy-8-ribityllumazine (photolumazine III)	Fragments of the detected ion at *m/z* 428.1197 ([M-H]^-^) from recombinant MR1 expressed in insect cells with *M. smegmatis* (mc^2^155) or *E. coli* infection match the fragmentation pattern of synthetic compounds with unclear accuracy.	hpMR1 cells	TRAV1-2^+^ and TRAV1-2^-^ clones activated	↑IFN-γ	Strong activation of MAIT cell TRAV1-2^+^ clone (D481C7) compared to diverse MR1T cell TRAV1-2^-^clone (D462E4).	(43)
6-(2-carboxyethyl)-7-hydroxy-8-ribityllumazine (photolumazine I)	Fragments of the detected ion at *m/z* 385.0994 ([M-H]^-^) from recombinant MR1 expressed in insect cells with *M. smegmatis* (mc^2^155) infection match the fragmentation pattern of synthetic compounds with unclear accuracy.	hpMR1 cells	TRAV1-2^+^ and TRAV1-2^-^ clones activated	↑IFN-γ	Activation of MAIT cell clones (D481C7 and D481F12) and a TRAV1-2^-^ diverse MR1T clone (D462E4 with TRAV12-2^+^).	(43)
6-formylpterin (6-FP)	MS detection of *m/z* 193.03 ([M-H]^-^) and its fragments from recombinant MR1 refolded with folic acid (vitamin B9) match those of the synthesized. 6-FP is also a compound in cell culture media.	Human lymphoid C1R cells; Jurkat.MAIT TRAV1-2-TRAJ33 and TRBV6-1, TRBV6-4, or TRBV20 cells	↓CD69, IL-2; Upregulates MR1 surface expression on C1R cells; No Jurkat.MAIT cell activation.	↓IFN-γ, TNF-α	*(i) Competitive Inhibitor*; *(ii)* Stain various MR1T cells but only weakly activate some TRAV1-2^+^ MAIT cells (M33-64) and TRAV1-2^−^ (TRAV21^+^) diverse MR1T (MAV21)	(17, 41, 50)
Acetyl-6-formylpterin (Ac-6-FP)	No evidence of MS detection from media or cells. 6-FP synthetic analog to load MR1 tetramer and better block MAIT activation than 6-FP itself.	Human lymphoid C1R and C1R.RM1 cells; Jurkat.MAIT cells	↓CD69, IL-2; Upregulates MR1 surface expression on C1R and C1R.MR1 cells; No Jurkat.MAIT cell activation	–	*(i) Competitive Inhibitor*; *(ii)* Strongly inhibit Jurkat cells with MAIT TCR expression, *(iii)* stain various MR1T cells but only weakly activate some TRAV1-2^+^ MAIT cells (M33-64) and TRAV1-2^−^ (TRAV21^+^) diverse MR1T (MAV21)	(41, 50)
5-(2-Oxopropyl)-8-[(2S,3S,4R)-2,3,4,5-tetrahydroxypentyl]-1,5,7,8-tetrahydropteridine-2,4,6(3H)-trione (4)	Synthetic compound with enhanced stability compared to 5-OP-RU, No evidence of MS detection from media or cells.	Human MR1-expressing HeLa (HeLa.MR1) cells; TG40.MAIT-TCR cells	↑CD69, Upregulates MR1 surface expression on HeLa.MR1 cells		Activation of “pan-cancer” TG40.TCR^+^ MAIT lines	(51)
JYM72 (chemical name unknown)	Synthetic compound with enhanced stability compared to 5-OP-RU, No evidence of MS detection from media or cells.	Human MR1-expressing HeLa (HeLa.MR1) cells; B16F10 melanoma cells-JYM72 pulsed	Upregulates MR1 surface expression on HeLa.MR1 and CR1.MR1 cells	↑ IFN-γ, TNF	*(i)* JYM72 pulsed THP-1 cells induces elevated Th1 cytokine production. *(ii)*JYM72 pulsed B16F10 melanoma cells enhances antitumor immunity by modulation of NK cell activity. *(iii)* JYM72 with CpG transfer to mice increases MAIT cell accumulation in the lungs.	(45, 52)
5-OP-RU analog (hydroxyethylamine)	Synthetic compound, No evidence of MS detection from media or cells.	Mouse MR1-overexpressing NiH.cl9 cells	↑CD137, Upregulates MR1 expression		Induction of MR1 surface expression and MAIT cell activation similarly to 5-OP-RU	(53)
5-OP-RU analog (propylamine)	Synthetic compound, No evidence of MS detection from media or cells.	Mouse MR1-overexpressing NiH.cl9 cells	↓CD137		Concentration-dependent inhibition of 5-OP-RU MAIT cell activation	(53)
Pyridoxal (PL)	Leukemia, Breast cancer, melanoma, lung cancer	C1R.MR1*01 cells	Upregulates MR1*01 cell surface expression	↑CD69	Activates a T cell line transduced with the 7.G5 TCR reported as a “pan cancer” receptor	(54)
Guanosine* and guanine adducts	Detected in MR1 eluted ligands from human melanoma cells under carbonyl stress or altered purine metabolism	THP-1 loaded guanosine or guanine; used cells under ADA inhibition to raise endogenous guanosine	↑CD69; Upregulation of IFN-γ production in guanosine-reactive MR1T cells (clone-specific)	↑ IFN-γ	MR1T cells selectively recognize guanosine-stressed tumor cells. *A candidate weak stimulator with evidence of MAIT stimulation and unknown MR1 binding capacity. Weakly activates clonal and polyclonal MAIT cells dependent on MR1 molecule.	(19, 20)
2-deoxy-5-formyluridine	Detected in melanoma tumor cells as ROS-oxidized thymidine derivative (5-FdU).	A375-MR1 melanoma cells pulsed with 5-FdU or treated with mitochondrial inhibitors	Upregulation of IFN-γ in 5-FdU-reactive diverse MR1T cells; MR1 surface expression depends on mitochondrial Complex III	↑ IFN-γ	Diverse MR1T cell clones (GP2A20, GP2A36) selectively recognize self-metabolites from metabolically altered cancer cells (ROS/high glycolysis).	(20, 30)
M_3_Ade (Carbonyl adduct of adenine)	MS detection of *m/z* 298.0931 ([M+H]^+^) from recombinant soluble MR1 expressed in A375 melanoma cells. Its fragments match the synthetic compound.	THP-1, A375-MR1, and melanoma cells pulsed with synthetic M1Ado; or treated to induce endogenous formation	Upregulation of CD69 and IFN-γ in diverse MR1T cells; selective activation of M1Ado-specific TCR clones	↑ IFN-γ	Potent stimulation of MR1-restricted T cells MR1-M_3_Ade tetramer reactive T cells were found in acute myeloid leukemia, cell lung adenocarcinoma, and hepatocarcinoma.	(18)
M1Ado (Carbonyl adduct of Adenosine)	MS detection of m/z 322.1143 ([M+H]+) from recombinant soluble MR1 expressed in A375 melanoma cells. The fragments of the base part (MS3) match the synthetic compound.	A375-MR1, THP-1, and melanoma cells pulsed with synthetic M1Ado or exposed to methylglyoxal to enhance endogenous adduct formation	↑CD69; Upregulation of IFN-γ in responsive diverse MR1T clones (M1Ado-reactive); tetramer staining shows specific TCR binding	↑ IFN-γ	MR1-M1Ado tetramer detected cells from lung cancer biopsies. Challenge with MR1-M1Ado-pulsed THP-1 cells induced IFN-γ production.	(19)

*guanosine as a weak stimulant without evidence of binding.

↑, increase; ↓, decrease.

These ribityl-pyrimidines are very unstable in aqueous acidic conditions and lead to the formation of the ribityl lumazine (RL) and 7-methyl-ribityllumazine (RL-7-Me), which present weak stimulatory activity and poorly upregulate MR1 surface expression. MAIT cells also recognize intermediate metabolites from the bacterial riboflavin pathway, such as 6,7-dimethyl-8-ribityllumazine (RL-6,7-diMe, a natural chromophore in lumazine protein) and 6-methyl-7-hydroxyl-ribityllumazine (RL-6-Me-7-OH), inducing weak stimulation of Jurkat cells with MAIT TCR overexpression and human MAIT cells from peripheral blood (17, 41, 48). These predicted and further synthesized ribityllumazine compounds match the detected mass-to-charge (*m/z*) unit of 329.11 from the *Salmonella typhimurium* culture media (17). As in **Table 1** and **Figure 3**, more ribityllumazine compounds, such as photolumazine I and photolumazine III, were detected from the recombinant MR1 protein expressed in *Mycobacterium smegmatis*-infected insect cells through matching collided fragment patterns with those of synthetic compounds (43). Both metabolites activated MAIT cell clones (e.g., D481C7 and D481F12 clones) and TRAV1-2^-^ MR1T clones (e.g., D462E4 with TRAV12-2) (43). More recently, pyridoxal (PL), another vitamin-related ligand, was identified in leukemia, breast cancer, melanoma, and lung cancer using mass spectrometry. Pyridoxal and pyridoxal phosphate have been reported to activate T cell lines transduced with the 7.G5 TCR, a receptor recently characterized for its ability to recognize MR1-presented ligands across a broad range of tumor types, including both hematologic and solid malignancies, thus demonstrating functional “pan-cancer” reactivity (54).

### Synthetic analogues

To evaluate the impact of the ribityl chain on MAIT cell activity, several 5-OP-RU analogs varying the 6-alkylamino substituents on the uracil were designed and synthesized (**Table 1**; **Figure 2**; **Figure 3**) (53). From these analogs, hydroxyethylamine induces MR1 surface expression and activates mouse MAIT cell line 6C2 cells comparable to 5-OP-RU stimulation, while the propylamine analog shows inhibition of 5-OP-RU-activated MAIT cells, similarly to AC-6-FP (53). To enhance MR1-ligand binding stability compared to 5-OP-RU and maintain MAIT cell activity, some vitamin-related synthetic analogs have been designed and synthesized, for example, the 2-oxopropyl pteridine (51) and JYM72 with an unknown chemical name (52) that display stimulatory activity for a murine T hybridoma cell TG40 and a human T cell line Jurkat expressing MAIT TCRs, respectively. Interestingly, B16F10 melanoma cells pre-pulsed with the MAIT cell antigen JYM72 have shown an enhanced antitumor immunity via an MAIT cell-modulated NK cell response (45) (**Table 1**; **Figure 3**). This is consistent with earlier findings that MAIT cells can promote NK cell activation and cytotoxicity within the tumor microenvironment, highlighting the potential for MAIT-NK crosstalk as a mechanism of antitumor immunity (45, 46) and the need for an appropriate stimulating strategy to induce protection (47).

### Polar self-metabolites

Cancer cells or mammalian cells-derived polar metabolites as agonists or antagonists for self-reactive MAIT and diverse MR1T cell activation can be closely linked to altered metabolic pathways in cancer, bridging cancer cell metabolism with immune regulation and surveillance. Recent discoveries indicate that physiologically relevant nucleobase and nucleoside compounds, as critical precursors for RNA synthesis and metabolism in mammalian systems, stimulate diverse MR1T cells via MR1-mediated antigen presentation (18–20). One such compound, 5-formyl-deoxyuridine (5-fdU), a modified nucleoside formed by oxidative damage to pyrimidine generated during cellular stress, was recently identified as an MR1 antigen capable of activating diverse MR1T cells (20, 30, 73). This result suggests the existence of diverse mammalian-derived polar metabolites that can modulate diverse MR1T cell responses, emphasizing the need to understand metabolite production and function within distinct metabolic pathways. Scientists from Switzerland recently demonstrated that carbonyl-nucleobase adducts (**Table 1**; **Figure 2**; **Figure 3**), including carbonyl adduct formation with adenosine (M_1_Ado), deoxyadenosine (M_1_dA), adenine (M_1_Ade), and guanine (M_1_Gua) with various activities for diverse MR1T cell activation, which may be accredited to upregulated metabolic pathways in cancer cells that generate high amounts of these identified carbonyl adducts (19). A subsequent study complemented these findings by additionally identifying a carbonyl nucleobase adduct of adenine (M_3_Ade), MR1-M_3_Ade loaded tetramers recognized heterogeneous MR1-reactive T-cells in healthy donors and patients with acute myeloid leukemia, and tumor-infiltrating lymphocytes from non-small cell lung adenocarcinoma and hepatocarcinoma *ex vivo* (18, 74, 75). These findings suggest that while the small molecules themselves are not unique to cancer cells, their elevated production under cancer-associated metabolic dysregulation may create a window for immune recognition. In culmination, these intriguing developments give rise to potential tumor-targeting strategies by using novel self-antigens, which occur in different metabolic pathways and are regulated by oxidative or carbonyl stress, to harness future TCR-based cancer immunotherapies and deepen the understanding of unconventional T cells in tumor immunity.

To comprehend metabolic pathways and metabolite profiles altered in cancer, research has indicated that cancer cell metabolism undergoes various transformations, such as a shift from oxidative phosphorylation to aerobic glycolysis (Warburg effect), or higher pressure of oxygen or carbonyl species, leading to changes in competitive nutrient utilization and metabolite prevalence compared to non-malignant cells (76, 77). Metabolic reprogramming throughout tumor progression stages is expected to be different between tumor regression and tumor progression stages. An early immune surveillance stage may be attributed to an effective anti-tumorigenic inflammatory microenvironment mediated by local immune cells and factors to inhibit tumor progression. However, a later cancer progression stage likely fosters an immunosuppressive landscape with a pro-tumorigenic microenvironment. In this context, the immune-dysregulated cancer microenvironment inherently impacts the availability and specificity of polar metabolites for MR1, which in turn critically influences MAIT cell activation downstream. Specifically, this metabolic shift may impact the production and loading of MR1 ligands, likely further regulating MR1 expression and effector function in the cancer microenvironment.

## MR1-restricted T cells in cancer

Self-reactive MAIT cells expressing an invariant Vα19 TCRα (TRAV1) chain were initially cloned and tested for their dependence on MR1-mediated antigen presentation in mice (11, 12, 34, 35, 40). In humans, MR1-restricted T cells exhibit high clonal diversity, characterized by variant TCR chains, the recognition of cancer cells, and the potential ability to kill cancer cells. Specifically, these MR1-restricted self-reactive human T cells from blood samples of healthy donors or cancer patients mostly express diverse TCRs (diverse MR1T or TRAV1-2^-^ MR1T) (13, 18, 44). A TRAV1-2^-^ MR1T clone (MC.7.G5), as an early example of antitumor diverse MR1T cells, is generated from the blood of healthy donors and is capable of cancer cell killing, via responding to a cancer instead of a bacterial metabolite (44). This diverse MR1T clone exhibits pan-cancer cytotoxicity to kill various types of cancer cells (44), but not a pan-population effect in humans, due to its restriction by an unusual MR1 allomorph with an R9H mutation in humans (78, 79). More diverse MR1T clones respond to cancer cells, dependent on the dominant MR1 allomorph in humans (13, 30). Many of these diverse MR1T clones are later shown to recognize nucleobase adducts with a carbonyl or aldehyde group (18, 19), which is potentially related to mitochondrial metabolic reprogramming with oxidative stress in melanoma and leukemia cell lines (30). The activation of diverse MR1T cells with self-metabolite stimulation induces the differentiation of memory subsets (18), demanding further understanding the dynamics and transcriptional programs of these diverse MR1T cells, compared with MAIT and conventional T cells.

In later years, MAIT and diverse MR1T cell responses in cancer immunity have been suggested to be highly dependent on context and influenced by the tumor microenvironment (TME), metabolic constraints, and immune-suppressive factors (13, 80). It is known that MAIT cells are enriched in mucosal tissues, including the lungs, liver, and intestines, and have been detected in tumor-infiltrating lymphocytes (TILs) across cancer types (81). Clinical research has reported that MR1 expression in tumors varies significantly, with some cancers upregulating MR1 as a potential immune evasion strategy, correlating with poor prognosis (72), while others exhibit reduced MR1 expression, potentially limiting MAIT and diverse MR1T cell activation (26, 82). Overall, MR1-restricted T cells display functional plasticity with tumor-suppressive or tumor-promoting responses across various malignancies, reflecting the dynamic influence of the tumor microenvironment (**Table 2**). Local factors, including cytokine signals, metabolic composition, and cellular interactions, influence MAIT cell activity, which supports either tumor control or tumor growth. This duality arises from their ability to produce inflammatory cytokines such as IFN-γ and TNF-α, contributing to tumor cell lysis, while also potentially promoting immune suppression through IL-17, IL-10, and regulatory interactions within the TME (83). These divergent outcomes depend on tissue context, disease stage, and ligand availability, and the mechanisms governing this dichotomy have been recently reviewed in greater detail (26).

**Table 2:** MAIT cell frequencies, responses, and functions in tumors.

Tumor type	MAIT frequency in blood	MAIT frequency in tumor tissues	MR1 expression	MAIT activation	Protumor response	Tumor immune modulation	Antitumor response	Ref.
AML	↓	?	↑mRNA	?	↓ IFN-γ, TNF-α ↑ IL-8	↑ PD-1 ↓ CD45RA-CCR7-	?	(55–57)
CLL	↓ CD26^hi^CD8^+^ T cell	?	↑L721.221, C1R, Jurkat, SupT1 cells	?	TIGIT^+^CD27^+^, ↓ CD107a	Gal-9/TIM-3	Granzyme B, Perforin ↑ CCR4^+^CCR6^+^, ↑ CCR6^+^CXCR3^+^	(58, 59)
MM	↓ (newly diagnosed, relapsed/refractory)	?	Present (Ac-6-FP upregulates)	MR1-dependent (5-OP-RU artificially pulsed APCs)	↓ IFN-γ (newly diagnosed), TNF-α	↑PD-1, ↓CD27	Retains CD161, IL-18Ra	(60, 61)
CRC	↓ (CD8+ memory, advanced CRC)	Normal or ↑	Present	MR1-dependent; IFN-γ driven	↓ IFN-γ (tumor site), ↑IL-17, IL-13	Th2 (IL-13) or Th17 (IL-17) skewed response	IFN-γ, TNF-α, perforin, granzyme B, Eotaxin; 5-A-RU activation kills cancer	(62–64)
Lung Cancer	↓ or ↑ (CD3+Va7.2+CD161+ cells)	?	?	?	↑ IL-6, IL-8 (serum)	↑ CD38	↑IFN-γ (serum)	(65, 66)
Breast Cancer	Normal	Present (epithelial ducts)	?	MR1-dependent; IL-17A-mediated	IL-17A (bacterial exposure)	IL-17A-biased response (Th17-skewed)	?	(67, 68)
HCC	Normal or ↓	↑	Present or ↓	MR1-dependent; TAM modulation	IL-8 (intratumoral)	CSF1R+PD-L1+ TAMs	↓ CSF1R^+^ TAMs restores cytotoxicity	(69, 70)
Glioma		CD8^+^, HLA-DR^`^, CD56^-^	Present	?	IL-17		TNF-α, IL-12	(71)
GBM	?		↑	MR1-dependent; co-stimulation via IL-12		?	↑ CD69, Ki-67, CD107a, IFN-γ, TNF-α (aAPC-expanded MAIT)	(39, 72)

?: Unknown or undetermined. Acute myeloid Leukemia (AML); Chronic lymphocytic leukemia (CLL); Multiple myeloma (MM); Colorectal cancer (CRC); Hepatocellular carcinoma (HCC); Glioblastoma (GBM).

↑, increase, ↓, decrease.

**Figure 4:**
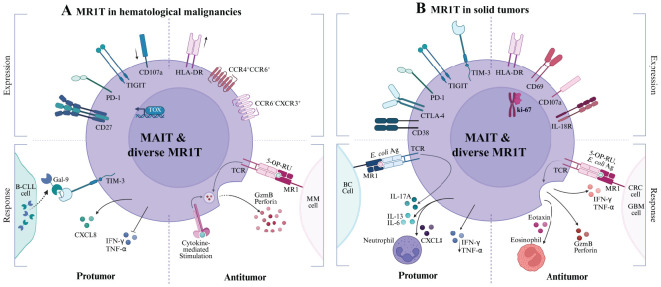
MR1T cell responses in Hematological Malignancies **(A)** and Solid Tumors **(B)**. Each panel is divided into quadrants to illustrate the relationship between MR1T cell phenotype (top, labeled “Expression”) and functional outcome (bottom, labeled “Response”). Top left quadrant: pro-tumor immunosuppressive or exhausted phenotypes. Bottom left quadrant: tumor-promoting cytokine profiles and dysfunctional responses. Top right quadrant: antitumor effector and activation phenotypes. Bottom right quadrant: tumor-controlling protective responses. Cancer cells depicted include **(A)** malignant B cell-chronic lymphocytic leukemia (B-CLL), multiple myeloma (MM); **(B)** Breast cancer (BC), colorectal cancer (CRC), glioblastoma (GBM).

However, whether the availability and identities of MR1 ligands regulate the levels of MR1 expression and shape the impact of MAIT cells across different cancers remains largely unknown, making their role in tumor immunity an area of active investigation. In hematological malignancies, such as multiple myeloma and leukemias, the role of MAIT cells remains largely underexplored, with limited clarity on whether MAIT cells contribute to tumor suppression or progression (**Table 2**; **Figure 4**). In solid tumors, MAIT cells exhibit more complex and variable functions, influenced by the tumor microenvironment, cancer metabolic program, local immune cell response, and immune checkpoint regulation (**Table 2**; **Figure 4**).

### Hematological malignancies

In acute myelogenous leukemia (AML), a disease marked by the uncontrolled proliferation of undifferentiated myeloid cells, MAIT cells show significant reduction in circulation among newly diagnosed patients, coinciding with elevated HLA-DR expression on MAIT cells, suggesting a recent activation state (56). Upon chemotherapy for 15 days in these AML patients (n=25), MAIT cell frequency is dramatically reduced to an average frequency of 3% of that prior to chemotherapy (56). A similar study analyzing a cohort of AML patients reveals similar alterations in MAIT cell frequency and function, correlating with disease burden and progression status. MAIT cells exhibit an upregulated PD-1 expression, a downregulated CD45RA^-^CCR7^-^ effector memory subset, impaired IFN-γ and TNF-α production, and begin favoring a cytokine profile (IL-8) linked to tumor progression (55). Notably, a lower MAIT cell frequency is independently associated with poorer patient prognosis, contrasting conclusions seen in other tumor types (56). To date, clinical data underscore the probable roles of MAIT cells adopting dysfunctional responses, potentially impacted by poorly understood metabolic and immune regulatory factors occurring in AML cells. For example, Tet methylcytosine dioxygenases 2 mutation promotes leukemogenesis likely through stabilizing methyl-5-cytosine for epigenetic regulation (84, 85) and affects nucleoside modification (84, 86).

A similar pattern of MAIT cell depletion is observed in chronic lymphocytic leukemia (CLL), where MAIT cell frequency is markedly reduced, particularly within the CD26^hi^ T cell population previously reported (58). Findings indicate that the CML microenvironment employs immune evasion modulation that promotes the apoptosis of CD8^+^CD26^hi^ T cells through the galectin-9 (Gal-9)/TIM-3 axis, leading to a pronounced depletion of this subset occupying the majority of MAIT cells (58). Interestingly, CD8^+^CD26^hi^ T cells enriched with MAIT cells also significantly reduce in CLL patients, and show a propensity to highly express cytotoxic molecules when stimulated with cytokines rather than CD3/CD28-dependent stimulation, indicating MR1 recognizing CLL-derived ligand is remiss but potentially important for MAIT cell activation in this cancer context.

In multiple myeloma (MM), a plasma cell malignancy thriving within an immunosuppressive bone marrow niche, MAIT cells are again depleted in circulation, along with a diminished frequency present in the BM (60), suggestive of depleted cell numbers not attributable to redistribution but rather dysregulation of MAIT cell differentiation and function within TME. Reduced frequencies are especially prominent in newly diagnosed and relapsed/refractory patients in recent observations (61). A defining feature of MAIT cells in MM is their upregulation of PD-1^+^ and CD27^+^, indicative of T cell exhaustion. *Ex vivo* and *in vivo* experiments demonstrate that blocking PD-1 signaling combined with α-GalCer-stimulated iNKT cells partially restores MAIT cell cytokine production (60), highlighting the potential for checkpoint blockade therapies in MAIT cell reactivation. Other research reveals MM cell lines exhibit detectable basal MR1 surface expression upon exposure to the folate-derived ligand 6-FP from the vitamin B9 pathway. These findings suggest that MM cells possess a reservoir of ER-resident MR1 capable of rapidly trafficking to the cell surface, where MR1 can bind or replace ligands for MAIT cell activation or inhibition. Additionally, MAIT cell-mediated cancer killing was apparent in MM cell lines pulsed with the potent 5-OP-RU agonist (61). This approach highlights the possibility that selective MR1 agonists can enrich MAIT cell-mediated tumor immunity in MM and may also prove relevant in other tumors; however, *in vivo* models are essential to examine the stimulatory molecules to induce an anti-cancer effect of MAIT cells.

### Solid tumors

Malignant gliomas, a type of gliogenic brain tumors, show evidence of MAIT cell tumor infiltration that displays an exhausted phenotype characterized by high expression of PD-1^+^, TIM-3^+^, and LAG-3^+^. Although MAIT cells in glioma tumors have been shown to express CD8^+^HLA-DR^+^, demonstrating a favorable activated phenotype, they lack CD56, a NK marker associated with enhanced MAIT cell responsiveness to Th1 cytokine stimulation (71). TRAV1-2^+^ TRAJ12/33 MAIT cells have been detected within brain tumor lesions, with MR1 expression identified in some cancerous glial cells. In glioblastoma (GBM), one of the most aggressive glioma malignancies, higher MR1 expression has been correlated with poor prognosis (72). While the mechanism remains unclear, this correlation raises the possibility that MR1-restricted MAIT cells may play a tumor-promoting role in GBM, or that elevated MR1 expression does not necessarily reflect increased presentation of stimulatory ligands capable of inducing protective MAIT responses. However, when expanded *ex vivo* and activated using artificial antigen-presenting cells, MAIT cells demonstrate strong cytotoxic potential against GBM cells via CD107a degranulation and lactate dehydrogenase detection. Upon activation, flow cytometric results illustrate elevated levels of IFN-γ and TNF-α, along with enhanced expression of CD69^+^ and Ki-67 activation and proliferation markers (39). MAIT cells have also demonstrated the ability to effectively lyse GBM cells in an MR1-dependent manner at higher effector-to-target ratios, underscoring their potential to target gliomas via MR1-antigen recognition and highlighting new avenues for glioma immunotherapy.

A similarly complex pattern emerges in lung cancer (LC), where MAIT cells exhibit various functions depending on disease stages and tumor microenvironmental factors. A clinical study investigating MAIT cell relevance in mucosal-localized tumors, inclusive of lung cancer, found reduced circulating MAIT cells, in contrast to elevated frequencies within tumor tissue. Strikingly, MAIT cells retain normal cytokine profiles with capacities for IFN-γ, IL-17, and TNF-α production (65). Conversely, another clinical study has observed CD3^+^Va7.2^+^CD161^+^ MAIT cells in circulation are significantly elevated in lung cancer patients, showing an activated pro-inflammatory state by CD38^+^CD8^+^ expression. Serum cytokine profile from this patient cohort reveals elevated IFN-γ, IL-6, and IL-8 production, suggesting that MAIT cells may contribute to shaping the inflammatory milieu (66). Further analysis shows that the IL-6 expression level correlated positively with tumor-associated MAIT cells frequently expressing CD38, leading to a possible immunosuppressive role. LC patients with an overall higher MAIT cell level, particularly CD38^+^CD8^+^ expression, are associated with worse progression-free survival, highlighting a detrimental role in lung cancer progression (66).

In colorectal cancer (CRC), MAIT cells exhibit both antitumor and protumor roles, with their function heavily influenced by the local inflammatory environment (64). Tumor-controlling potential of MAIT cells is generally associated with the increased tumor-infiltrating MAIT cells exhibiting a Th1 phenotype that secreted granzyme B and, to a lesser extent, perforin, suggesting a protective antitumor role in colon adenocarcinomas (87). Supporting this, RAG-/- mice bearing the murine colon adenocarcinoma cell line MC38-derived tumors demonstrate significant tumor growth inhibition when MAIT cells are injected peritumorally. Tumors treated with MAIT cells display elevated levels of pro-inflammatory cytokines (IFN-γ, IL-17, GM-CSF), and eosinophil-attracting chemokines (eotaxin-1), alongside increased caspase 3/7 activity, indicative of enhanced tumor cell death (62). Complementary *in vitro* experiments with human MAIT cells stimulated by 5-A-RU further demonstrate their capacity to kill COLO 205 cancer cells, enhance cytokine production, and promote eosinophil activation and recruitment, as evidenced by upregulated CD69^+^ and granzyme A expression (62). However, MAIT cells, characterized by impaired Th1 cytokine production or increased IL-17 secretion, adopt an immunosuppressive phenotype within the TME, which sustains chronic inflammation and tumor growth, or enhance IL-13 expression, fostering a Th2-skewed and protumor microenvironment (63, 64). This shift dampens effective cytotoxic responses while promoting tumor-associated inflammation and myeloid cell recruitment. Notably, IL-17-driven inflammation has been strongly linked to tumor progression, correlating with worsened CRC prognosis (63). Furthermore, decreased circulating MAIT cells, particularly within the CD8^+^ memory subset, have been associated with advanced-stage CRC. Whereas paradoxically, a higher tumor-infiltration of MAIT cells, detected in CRC tissues compared with that of non-tumor tissues (63), may modulate anti-cancer immunity and affect patient survival, potentially dependent on Th1 or Th17-like phenotype of the infiltrated MAIT cells. This duality underscores the need for further research into the regulatory mechanisms that dictate MAIT cell functional polarization in CRC. Understanding cancer cell metabolism, in particular cancer metabolite profiles for MAIT cell activation or inhibition, may reveal new therapeutic strategies aimed at modulating MAIT cells to enhance antitumor immunity while limiting their protumor activities.

MAIT cells in hepatocellular carcinoma (HCC) underline their functional plasticity, with studies presenting conflicting evidence regarding their impact on prognosis. Research suggests that a higher abundance of MAIT cell infiltrates correlates with improved patient outcomes (88), while other clinical studies indicate that elevated intratumoral MAIT cells are associated with poor prognosis (70). This study further demonstrates that intratumoral MAIT cells from HCC patients upregulate the expression of PD-1^+^, CTLA-4^+^, and TIM-3^+^ inhibitor or exhaustion markers along with diminished cytotoxic molecules, including IFN-γ, granzyme B, and perforin, in comparison to MAIT cells from peritumor regions. Within a detrimental TME, infiltrating MAIT cells display an exhausted phenotype, largely driven by tumor-associated macrophages (TAMs), which induce dysfunction through increased expression of CSF1R^+^ (colony stimulating factor 1 receptor), PD-L1^+^ (programmed cell death ligand 1), and CD69^+^. This direct cell-cell interaction suppresses MAIT cell activity, leading to exhaustion and the loss of cytotoxic function (69). Via paracrine regulation, the presence of IL-8 within the HCC microenvironment indicates MAIT cell dysfunction via inhibiting IFN-γ production (70). More recent findings reveal that HCC patients exhibit a significant reduction in circulating MAIT cells alongside limited infiltration into liver tumors. Despite these immunosuppressive mechanisms, lower levels of MAIT cell infiltration have been linked to a worse prognosis, suggesting that MAIT cells may contribute to tumor control if their functional integrity and protectivity can be maintained, and they are not pushed into a chronically exhausted state. Notably, murine HCC models have demonstrated that depletion of CSF1R^+^ TAMs improves MAIT cell infiltration and restores cytotoxic function (69), highlighting the potential of targeting the MAIT cell-TAM axis as a promising strategy to enhance immunotherapy responses in HCC. Current findings emphasize the need for further investigation into strategies that can preserve MAIT cell functionality while preventing their immunosuppressive conversion within a cancerous microenvironment of the liver.

In the breast cancer context, MAIT cells are primarily retained in circulation but are also detectable within the epithelial ducts of human breast tissue. Gene transcriptomic analysis has identified MAIT cell-specific markers, including TRAV1-2^+^ TCR, CD161, PLZF, and IL-18Rα, within the epithelial ducts, suggesting their presence and potential functional role in breast TME (67). Notably, these tumor-associated MAIT cells exhibit a Th17-skewed functional profile, characterized by an enrichment of IL-17A-producing cells, the MAIT17 subset. This IL-17A bias is particularly significant, as IL-17A-mediated inflammation has been implicated in promoting tumor progression through the recruitment of protumor immune cells and the establishment of a chronic inflammatory milieu. Further *in vitro* experiments demonstrate that when MAIT cells are activated by *E. coli* in an MR1-dependent manner and co-cultured with breast cancer cell lines, they predominantly produce IL-17A while exhibiting a diminished Th1 or cytotoxic response (67). This shift away from IFN-γ and TNF-α production suggests that MAIT cells in the breast cancer microenvironment may contribute to tumor-promoting inflammation rather than effective antitumor immunity. Additionally, research has shown that MR1-restricted TRAV1-2^+^ and TRAV26-1^+^ TCRs identified from the tumor-infiltrating T cells of breast cancer patients specifically respond to some breast cancer cell lines but not to other cancer types (68). This observation implies a degree of antigen-specific recognition of breast cancer cells, highlighting the potential for tumor-selective immune interactions mediated by MR1. However, which breast cancer cell-derived metabolites bind to MR1 for MAIT cell activation and whether this selective recognition can be leveraged for therapeutic intervention or if it predominantly contributes to tumor immune evasion remain open questions, necessitating further exploration into the functional dynamics of MAIT cells in breast cancer.

Overall, the function of MAIT cells in cancer is shaped by the interplay between cancer metabolism, immune checkpoint regulation, and local cytokine signaling. In some settings, MAIT cells exhibit potent tumor control, whereas in others, they are co-opted into tumor-promoting activities. Understanding the mechanisms governing this functional plasticity is critical for designing effective immunotherapies that harness their tumor-killing potential capacities while mitigating their protumor advancements. Strategies aimed at reactivating exhausted MAIT cells, modulating MR1 antigen presentation, minimizing protumor MAIT cell subsets, and leveraging cytotoxic MAIT subsets or capabilities represent promising avenues for future cancer treatment.

## CD1-restricted T cells in cancer

CD1 molecules are nonpolymorphic in humans and present lipid-based antigens to unconventional T cells. Unlike classical MHC molecules using shallow hydrophilic ligand-binding grooves for peptide antigen presentation (**Figure 1**) (1, 2), CD1 molecules possess hydrophobic antigen-binding clefts and present lipid-biased ligands, providing a distinct mechanism for immune recognition (7, 14, 89). Studies demonstrated that CD1 molecules present mammalian cell-derived and tumor-associated lipids, such as gangliosides, phospholipids, and sphingolipids. CD1-mediated lipid antigen presentation permits CD1-restricted T cells to become lipid metabolite sensors, detect lipid metabolic alteration in cancer cells, and induce cancer immune surveillance and metabolic modulation. Translationally, lipid antigen presentation through CD1 may serve as a valuable target for immunotherapeutic strategies aimed at harnessing the antitumor potential of CD1-restricted T cells. CD1 proteins with lipid antigen-presentation functions consist of four isoforms in humans: CD1a, CD1b, and CD1c as group 1 CD1 proteins, and CD1d as a group 2 CD1 protein (8, 89, 90). Tumor cells manifest an altered lipid metabolism, leading to the overrepresentation of specific lipid antigens that may be loaded to CD1 proteins for the recognition of CD1-restricted T cells. Advanced by mass spectrometry-based lipid profiling, recent discoveries demonstrated that CD1 proteins bind various classes of heterogeneous mammalian cell-derived lipids by groups I and II human CD1 proteins (20, 89, 91–94). CD1a binds skin-derived lipids (95), implying a potential involvement in cutaneous malignancies. CD1b has been shown to bind to tumor-derived phospholipids in T-cell lymphoma (96). CD1c recognizes methyl-lysophosphatidic acid (97), a lipid abundantly expressed in leukemic cells. Perhaps due to the first characterized invariant αβ T cell population called invariant natural killer T (iNKT, type I NKT) cells and the availability of lipid-preloaded CD1d tetramers for staining iNKT cells, CD1d has been particularly well studied for its role in presenting lipid antigens to iNKT cells (23, 98) and later shown to activate diverse NKT (dNKT, type II NKT) cells as well (99, 100).

The iNKT cells express a highly conserved TCR, comprising TRAV10 or TRAJ18 α-chain with limited variable β-chain such as TRBV24 usage (8), which recognizes α-GalCer purified originally from the marine sponge, Agelas mauritianus. Type I iNKT cells are known for their rapid cytokine responses upon activation, producing IFN-γ and TNF-α, which enhance antitumor immunity by recruiting and activating dendritic cells (DCs), NK cells, and CTLs. The iNKT cells also possess the capacity to exert direct cytotoxic effects on tumor cells via perforin, granzyme B, and FasL-mediated apoptosis (101). Given their pivotal role in modulating immune responses, CD1-restricted T cells have emerged as attractive candidates for therapeutic intervention. Several strategies have been explored to harness iNKT cells in cancer therapy (102), including lipid-based vaccines such as α-GalCer, which potently activate iNKT cells and induce robust Th1-skewed antitumor responses (103). Modified α-GalCer derivatives and CD1d-binding glycolipid agonists have been developed to improve cytokine bias and overcome iNKT cell anergy (104, 105). Advances in cellular therapies include chimeric antigen receptor (CAR)-NKT cells, which combine innate tumor-homing capabilities with engineered antigen specificity and have demonstrated enhanced cytotoxicity and persistence in preclinical lymphoma and melanoma models (106–108). Additionally, dendritic cell-based lipid vaccines have shown promise in expanding functional iNKT populations and boosting antitumor immunity (109). The balance between Type I and Type II iNKT cell activity influences the overall immune response within the TME. Type II iNKT cells exhibit TCR diversity and respond to a broader range of lipid antigens (8). While Type I iNKT cells largely promote antitumor immunity, Type II iNKT cells can occupy an immunosuppressive functionality, modulating immune responses through the production of IL-13 and TGF-β, which promote regulatory T cell expansion and limit effective immune responses. In multiple myeloma (110), Type II iNKT cells have been implicated in suppressing effective immune responses by inducing myeloid-derived suppressor cells (MDSCs) that modulate immune cells toward a suppressed, regulatory state (23). As the roles of CD1-restricted T cells in cancer immunity and therapeutic trials based on lipid antigens have been comprehensively reviewed (90, 111, 112), we focus on discussing the structures and functions of lipid metabolites as CD1 ligands in cancer immunity.

## Self lipid metabolites as CD1 ligands

**Figure 5:**
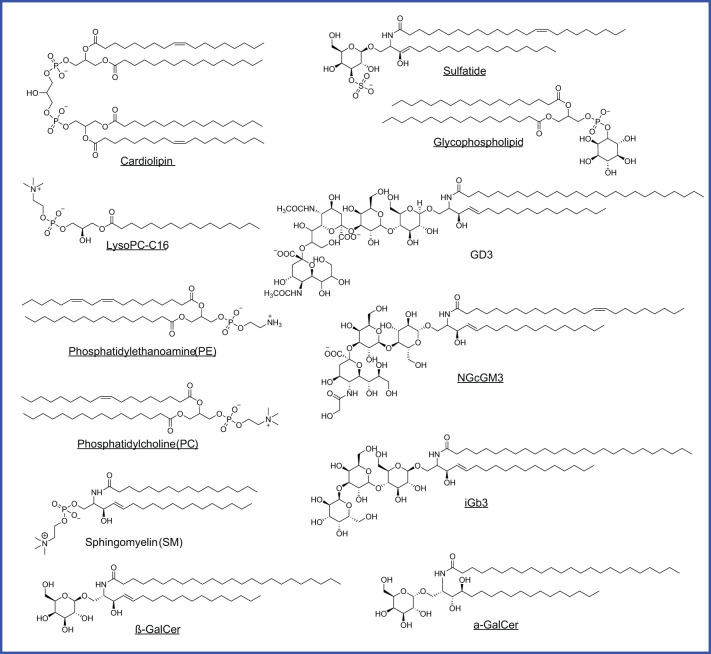
CD1d-bound lipids detected in different cancers. Agonists are underlined. Glycophospholipid is identified from HLA class I deficient human lymphoblastoid, MS detection with m/z 861.8 of [M-H]-, but with an unknown hexosyl group.

As CD1-loaded lipids likely bridge cancer cell lipid metabolism with T cell sensing of lipids, cellular lipids loaded to CD1 proteins are particularly interesting to be identified using tandem mass spectrometry in multiple studies. Two major structural categories of tumor-associated lipids, phospholipids and sphingolipids with or without glycosylated modification (**Table 3**; **Figure 5**), are sampled by human CD1d protein and function as agonists or antagonists for NKT cell responses (15, 20, 89, 91–94). Multiple classes of CD1-sampled lipids, including cardiolipin, sphingomyelin, sulfatide, and ganglioside, have been detected in tumor tissues by MS imaging from human glioblastoma (113).

**Table 3:** CD1d ligands detected from cancer cells activate or block CD1d-restricted T cell responses.

CD1d ligands	T cells	Detection in cancer or other cells	T cell activation	Effector molecules	Anti-cancer T cell immunity	Refrence
Cardiolipin (CL)	NKT & γδ T cells	Elevated in HCC murine hepatic cells; Identified from Corynebacterium glutamicum; detected by MS imaging in human glioblastoma	Dependent on acyl structure (16:1 activates NKT cells)	↑IFN-γ, CCL5	CL increases oxidative phosphorylation, supports tumor cell growth or mitochondrial degradation, leading to CL CD1d presentation (HCC).	(15, 92, 111–115)
Lysophosphatidylcholine (LPC) LysoPC-C16	dNKT (Type II)	Identified from myeloma patient plasma. Shown elevated levels in HCC murine hepatic cells via lipidomic analysis.	C18:1 LPC more binding to CD1d dimer than C16:1 LPC	↑ IL-13	High lipid profile associated with severe HCC & tumor evasion/anti-inflammatory cytokine profile	(110, 112, 114, 116, 117)
Phosphatidylethanolamine (PE)	NKT & γδ T cells	–	Cloned γδ T lymphocytes recognized pollen-derived 16:0/18:2 and 18:2/18:2 PE.	↑IFN-γ, IL-4, IL-13	Tumor-associated lipid/activates γδ T cells producing IFN-γ or regulatory cytokines.	(118–120)
Sphingomyelin	NKT	Upregulated in murine breast cancer cell lines and human CRC SW480 cells. Identified from ligand bound-CD1a^+^ HEK293 cells, detected by MS imaging in human glioblastoma	Blocks CD1d-lipid loading to inhibit iNKT activation	–	Elevated in early tumorigenesis; lyso-sphingomyelin is a weak agonist.	(93, 95, 113, 114, 121–123)
Phosphatidylcholine (PC)	NKT & γδ T cells	Identified from ligand bound-CD1a^+^ HEK293 cells.	Cloned γδ T cells are stimulated by natural and synthetic 16:0/16:0 PC	↑IFN-γ, IL-4	Cloned γδ T cells activation via C1R lymphoblastoid antigen-presenting cells.	(119, 120)
Glycosphingolipids (GSLs) iGb3	NKT	Synthetic	iGb3 and chemically modified 4‴-dh-iGb3 stimulate NKT cells	↑IFN-γ, IL-4	iGb3-loaded dendritic cells exhibit Th1 response and inhibit subcutaneous melanoma growth and limit lung metastasis in C57BL/6 mice.	(124)
Ganglioside NGcGM3	iNKT	Synthetic	NGcGM3 loaded CD1d drives iNKT activation	↑Ki-67 expression	Tumor-restricted lipid *in vitro* promotes and activates human iNKT cells via CD1d presenting B cells.	(125)
Ganglioside GD3	NKT	Synthetic and detected by MS imaging in human glioblastoma	GD3-reactive NKT cells show CD1d-restricted activation	↑IFN-γ, IL-4, IL-10	Mice immunized with human melanoma cells (SK-MEL-28) or GD3-loaded APCs stimulate iNKT response.	(113, 126)
Ganglioside Gg3Cer	NKT	From T cell lymphoma (L5178Y-R) and detected by MS imaging in human glioblastoma	Inhibitory ganglioside for CD1d-mediated NKT cell activation	–	Exogenous Gg3Cer exposure to murine CD1d^+^ fibroblasts inhibits iNKT activation	(111, 113)

Multiple classes of mammalian lipids, including ceramide, monohexosylceramide, dihexosylceramide, deoxy-dihydroceramide, sphingomyelin, triacylglycerol, phosphatidylcholine, lyso-phosphatidylcholine, ether-phosphatidylcholine, phosphatidylethanolamine, lyso-phosphatidylethanolamine, ether-phosphatidylethanolamine, and cardiolipin, have been extracted from CD1a, CD1b, CD1c, CD1d proteins expressed in human myelogenous leukemia K562 cell line and human embryonic kidney 293T cell line (9). Group I CD1 proteins, including CD1a (127), CD1b (128), and CD1c (125), can bind endogenous phospholipids and glycolipids to activate γδ T cells and are specifically included in this table.

↑, increase.

### Phospholipids

Phospholipids, as generally defined by a glycerol backbone linked to fatty acids and a phosphate-containing head group, are key components of cellular membranes and signaling pathways. Within this class, cardiolipin (CL), overexpressed early in hepatocellular carcinoma (HCC) in mice, is released during mitochondrial degradation and is associated with CD1d lipid presentation to iNKT and γδ T cells, promoting tumor growth by supporting mitochondrial function (114, 115, 120). Lysophosphatidylcholine (LPC), elevated in mouse HCC and multiple myeloma patients, fosters a tumorigenic lipid profile and immune evasion by activating anti-inflammatory NKT cells (110, 112, 116). Similarly, phosphatidylethanolamine (PE) and phosphatidylcholine (PC) (**Table 3**; **Figure 5**), both abundant membrane phospholipids, are presented by CD1d and CD1c to activate iNKT and γδ T cells, promoting either regulatory or Th1 cytokine responses (118, 119).

### Sphingolipids

Sphingolipids, in contrast, are built on a sphingosine base and often incorporate ceramide backbones, playing essential roles in membrane stability and signal transduction. Tumor-elevated sphingomyelin (**Table 3**; **Figure 5**), a sphingolipid enriched in the mammalian plasma membrane, has been shown to weakly stimulate iNKT cells through a mono-acylated derivative, whereas the di-acylated form is predominantly reported to act as an inhibitor when presented on CD1d (92, 93). Sphingomyelin also displays as an iNKT antagonist and contributes functionally to immune dysregulation across mouse breast cancer cells alongside human colon and B cell cancers (120–122). Glycosylated sphingolipids (GSLs), particularly isoglobotrihexosylceramide (iGb3), are a class of glycolipids containing amino alcohol sphingosine that have been shown to promote antitumor Th1 responses by activating iNKT cells to produce IFN-γ (124). Gangliosides, such as GD3, NGcGM3, Gg3Cer (**Table 3**; **Figure 5**), and salic-acid containing GSLs enriched in certain malignancies, can either inhibit or promote iNKT activation in a context-dependent manner, acting as tumor-specific antigens or immune modulators (125, 126, 129, 130). Although identifying CD1-loaded endogenous lipids (**Table 3**; **Figure 5**) has offered a critical example and framework for understanding intracellular metabolite loading for unconventional T cell activation, only a small list of endogenous ligands has been discovered, and their functional roles in cancer remain largely unexplored.

## The γδ T cells in cancer

**Figure 6:**
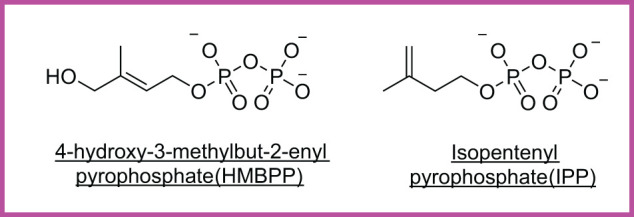
Cancer-associated phosphoantigens (pAgs). HMBPP, a microbial metabolite, and IPP, a host-derived mevalonate intermediate, are presented by butyrophilin proteins to activate Vγ9Vδ2 γδ T cells.

The γδ T cells represent a distinct arm of the T cell lineage, defined by their TCR expression of γ and δ chains, as opposed to the αβ TCRs found on other invariant T cell subsets and conventional T cells. Although γδ T cells can be activated by nonclassical MHC molecules and metabolite antigen complexes, γδ T cells can also recognize other metabolites or protein ligands, such as phosphoantigens. Briefly, Vδ1^+^ cells are more prevalent in mucosal and epithelial tissues; Vδ2^+^ γδ T cells, frequently paired with the Vγ9 chain, are the most abundant subset in peripheral blood; while Vδ3^+^ cells are less common and enriched primarily in the liver and gut (25). In TME, γδ T cells exhibit dual tumor responses, depending on their subsets, cytokine profiles, and tissue microenvironments. Vδ2^+^ cells generally contribute to antitumor immunity by secreting IFN-γ and TNF-α and inducing tumor cell lysis through secreting perforin, granzyme B, and TRAIL. Their expression of NKG2D enables direct recognition and killing of stressed or transformed cells expressing MICA/B and ULBPs (25). Studies have shown an increased infiltration of Vδ2^+^ TILs correlating with improved patient survival in malignancies such as malignant melanoma, AML, and ALL (145, 146). Other research suggests Vδ5^+^ or TRDV5^+^ γδ T cells that recognize EPCR demonstrate potent tumoricidal activity (147). On the other hand, Vδ1^+^ γδ T cells have demonstrated a more complex role. While capable of effective cytotoxicity and IFN-γ production, certain Vδ1^+^ populations secrete IL-17A, a cytokine associated with angiogenesis, neutrophil recruitment, and the promotion of MDSCs (134), all of which contribute to tumor progression. In rectal cancer, patients with increased Vδ1^+^ cell infiltration indicated higher tumor burden, whereas Vδ2^+^ cell presence exhibited a negative association with tumor size (135). Beyond this basic outline of important subsets of γδ T cells, we will briefly describe the roles of metabolite and protein ligands in anti-cancer γδ T cell responses (**Figures 5**, **6**; **Table 4**), as immune responses and therapeutic effects of γδ T cells have been reviewed recently (22, 25, 134).

**Table 4:** Unconventional T cells in cancer immunity.

Category	MR1-restricted T cells (MR1T)	CD1-restricted T cells	γδ T cells
TCR Subsets	MAIT or invariant MR1T with Vα7.2 (TRAV1-2^+^), and diverse MR1T (or TRAV1-2^-^ MR1T) with diverse TCRs	CD1d-restricted T cells are called NKT cells. invariant NKT (iNKT, type I): Vα24 (TRAV10^+^); diverse NKT (dNKT, type II): variable TCR	Vδ1+, Vδ2+, Vδ3+
Antigens (Stimulatory molecules for γδ T Cells)	Polar metabolites	Lipid metabolites	Phosphoantigen, lipid metabolites, stress protein ligands
Microbial or non-self antigens or ligands	Riboflavin (B2) precursors and their derivatives, folic acid (B9) derivatives, pyridoxine (B6) derivatives	α-GalCer, bacterial glycolipids, glycerolipids, sphingolipids, polyketides	α-GalCer
Antigen presentation molecules or protein ligands	MR1	CD1a,b,c (Group 1 CD1) CD1d (Group 2 CD1)	CD1a, b, c, d, BTN, MR1 for Vδ3+ (131) and Vδ2+ γδ T (132), MICA/B, ULBPs, and EPCR
Protumor effectors	MAIT17	Type II	Vδ1+ (rectal cancer)
Protumor mediators	IL-8, IL-17A	IL-13 (II)	IL-17
Protumor markers	↑CD39, CTLA-4, PD-1, ↓IFNγ	↑PD-1, ↓IFNγ	↑IL-17, ↓IFNγ
Tumor-induced immunosuppression	↑TAMs (CSF1R^+^), immune suppression	↑ Treg via IL-13, neutrophil recruitment	MDSC & neutrophil recruitment, angiogenesis
Antitumor effectors	MAIT1	Type I	Vδ2+, Vδ1+
Antitumor mediators	IFNγ, TNFα, IL-2, IL-17A, Perforin, Granzymes	IFNγ, IL-4, IL-10, IL-13, IL-17, IL-21, IL-22, Perforin, Granzymes, FasL, TRAIL	IFNγ, TNFα, TRAIL, Perforin, Granzymes
Antitumor responses	Dendritic cell/NK cell activation, rapid response, cytotoxicity	Activate APCs, remodel TME, cytotoxicity	Th1 responses, αβ T cell/APCs activation
References	(26, 83, 133)	(23, 101, 110)	(25, 134, 135)
Protective effects in clinical trials (CAR-T trials)	High cytotoxicity with low IFNγ production against lymphomas and breast cancer cells with CD19/CAR-MAIT (136)	*Representative trials*: ***(i)*** CD1d-restricted CAR19-NKT eradicate brain lymphomas & elevated cytotoxicity against CD19+ CLL cells (106); ***(ii)*** CSPG4-specific CAR-NKT kill melanoma *in vitro* with improved cytotoxicity (107); ***(iii)*** CD62L-CD19 CAR-NKT complete B-cell lymphoma regression in mice (108)	*Representative trials*: ***(i)*** CD19/GD2 CAR- γδ lysed malignant B cell and neuroectodermal cell lines showing enhanced cytotoxicity with CD69 and IFNα upregulation (137); ***(ii)*** γδ CD19 CAR-iT inhibited NALM-6 B cell lymphoma tumor growth and lysed cells *in vitro* and *in vivo* (138)
TCR-T based on invariant TCR chains	***Advantages***: ***(i)*** Recognize a wide range of tumors irrespective of HLA type, ***(ii)*** Limited expression on healthy tissues reduces off-target effects, ***(iii)*** Broad tissue distribution ***Disadvantages***: ***(i)*** Early stage of research, ***(ii)*** Need for identification of specific tumor-associated ligand *Representative trials*: None *Clinical Potential*: high (80, 139)	***Advantages***: Potential to modulate tumor microenvironment ***Disadvantages***: ***(i)*** Limited persistence *in vivo,* ***(ii)*** Potential for inducing anergy *Representative trials*: Phase study evaluated agenT-797, an unmodified, allogeneic iNKT cell therapy, in patients with moderate-to-severe ARDS secondary to SARS-CoV-2. The therapy was well-tolerated with preliminary evidence of efficacy. *Clinical Potential*: moderate to high (101, 140)	***Advantages***: ***(i)*** MHC-unrestricted tumor recognition, ***(ii)*** Potential for allogeneic use ***Disadvantages***: ***(i)*** Heterogeneous population, ***(ii)*** Limited understanding of antigen specificity *Representative trials*: Phase I trial assessed GDX012, allogeneic Vδ1+ γδ T cells, in patients with AML. The study demonstrated safety and potential antitumor activity. *Clinical Potential*: very high (141, 142)
Antigen-directed therapies	Unknown candidate ligand: ***(i)*** Broad patient applicability via MR1 (limited-polymorphism); ***(ii)*** MR1 is upregulated in metabolically stressed/cancerous cells; ***(iii)*** MR1 ligand stability (e.g., 5-OP-RU) is limited; ***(iv)*** tumor-specific MR1 presentation not fully characterized (80).	αGalCer: ***(i)*** Rapid cytokine production (IFNγ) upon activation, ***(ii)*** Potential to enhance antitumor immunity, ***(iii)*** used in vaccine strategies, ***(iv)*** Risk of inducing NKT cell anergy with repeated stimulation, ***(v)*** Limited persistence and expansion *in vivo*, ***(vi)*** Clinical trials exploring α-GalCer-pulsed dendritic cells (143, 144)	Phosphoantigens: ***(i)*** MHC-independent tumor recognition, ***(ii)*** Broad reactivity against tumor cells, ***(iii)*** Variable expansion and persistence in patients, ***(iv)*** Possible pro-tumorigenic effects in certain contexts (141)

Agonist and antagonist metabolites for unconventional T cells have been detailed in **Figures 2** and **3** (**Table 1**), **Figure 5** (**Table 3**), and **Figure 6**. Bold numbering is to separate various features. Butyrophillin (BTN); α-GalCer: α-GalactosylCeramide; MHC class I chain-related protein A and B (MICA/B); UL16-binding proteins (ULBPs); Endothelial protein C receptor (EPCR); Tumor associated macrophages (TAMs); Colony stimulating Factor 1 receptor (CSF1R^+^); Cytotoxic T-lymphocyte associated protein 4 (CTLA-4); Programmed cell death protein 1 (PD-1); Fas ligand (FasL); TNF-related apoptosis-inducing ligand (TRAIL); Chimeric antigen receptor-mucosal associated invariant T cell (CAR-MAIT); CD19^+^ chimeric antigen receptor-natural killer T cell (CAR19-NKT); Chondroitin sulfate proteoglycan 4 (CSPG4)-specific CAR-NKT; CD19^+^ B cell protein and disialoganglioside glycolipid tumor antigen (CD19/GD2 CAR); CD19 CAR-induced T (iT) cells.

## Stress-induced metabolites for γδ T cell activation

The γδ T subsets interact with protein ligands or metabolite antigens and shape their roles in immune surveillance and tumor immunity. A key feature of γδ T cells is their ability to recognize a broad array of stress-induced metabolites and tumor-associated metabolites not necessarily via antigen processing or presentation of MHC-like proteins (22, 25), which can be classified based on different Vδ chain expression. Different Vδ1^+^ T cell clones have demonstrated the ability to recognize respective lipid antigens presented by human CD1 proteins, including various endogenous phospholipids and glycolipids presented by CD1a (127), CD1b (128), and CD1c (125), as well as CD1d loaded with α-GalCer and sulfatide (28) (**Table 4**; **Figure 5**). Vδ1^+^ T cells can also be sorted using MR1 tetramers loaded with 5-OP-RU (148) and can differentiate with the stimulation of HLA-A2 from hematopoietic stem or progenitor cells (149). In other cases, Vδ1^+^ T cells interact with protein stress ligands such as non-classical MHC class I proteins, MICA/B and ULBPs, via Vδ1^+^ T cell surface NKG2D receptor, and annexin A2, a molecule linked to tumor cell stress and apoptosis (150, 151). Vδ1^+^ cells also engage ligands like heat shock proteins (HSPs) and the endothelial protein C receptor (EPCR) (147), which are upregulated under cellular stress. Functionally, Vδ1^+^ cells are shown to be dysregulated in malignancies such as colorectal cancer (152), or a candidate target of immunotherapy against neuroblastoma (153).

Vδ2^+^ T cells, represented by Vγ9Vδ2 T cells, the dominant circulating subset in humans, are activated by non-peptidic phosphorylated intermediates of the mevalonate pathway, collectively known as phosphoantigens (pAgs) (**Figure 6**), which are often upregulated in transformed or infected cells (25, 154). These cells require engagement of the butyrophilin family members BTN3A1 and BTN2A1, which cooperatively mediate γδ TCR activation (155). Recent structural studies have revealed that BTN2A1 binds to the lateral surface of the γδ TCR, leaving the apical region accessible for co-engagement by a second ligand in a BTN3A1-dependent manner. BTN2A1 and BTN3A1 also directly interact in cis to form multimeric W-shaped complexes, and this coordinated interaction is critical for full γδ TCR activation (156). Vδ2^+^ cells have also been shown to respond to human MutS homologue 2 (hMSH2) (157), a DNA repair protein aberrantly expressed on tumor cells, as well as bacterial superantigens and certain microbial-associated proteins (21, 158). Recent work has further expanded the functional scope of this subset by identifying a population of MR1-autoreactive Vγ9Vδ2 T cells that recognize MR1-self-antigen complexes in a butyrophilin-independent, CDR3δ-dependent manner, revealing a novel avenue for γδ T cell involvement in antigen-specific immunity (132).

Differently, a Vδ3^+^ T cell clone has shown ability to sense cell stress factors and recognize annexin A2 that binds to cell surface lipids (151) on tumor cells responding to stress and depending on the induction of reactive oxygen species. Additionally, processed insulin and Igλ light chains of host origin have been reported to stimulate γδ T cells in multiple myeloma and insulinoma (159, 160). These diverse recognition mechanisms underscore the capacity of γδ T cells to serve as versatile sentinels in tumor detection.

Recent studies have revealed variable roles for γδ T cell subsets across different cancer types. In human breast cancers, tumor-infiltrating Vδ1^+^ T cells have been associated with improved prognosis and are enriched in the tumor epithelium, where they exert cytotoxic capacity against cancer cell lines (161). Conversely, in glioblastoma and certain lung cancers, γδ T cells have been shown to skew toward an IL-17-producing phenotype, which may promote tumor progression by enhancing angiogenesis and recruiting myeloid-derived suppressor cells (162, 163). In hematological malignancies such as leukemia and lymphoma, circulating Vγ9Vδ2+ T cells often exhibit cytotoxic activity and are being investigated for adoptive immunotherapy (134). Clinical trials are underway evaluating the safety and efficacy of expanded or engineered γδ T cells, though challenges remain in ensuring their persistence, tumor infiltration, and avoidance of exhaustion within the tumor microenvironment. For a more comprehensive overview of γδ T cell antitumor effector responses and therapeutic trials, please refer to the recent reviews by Arias-Badia et al., Hayday et al, and Schoünefeldt et al. (22, 25, 134), as we focus on discussing the activation of γδ T cells and its association with cancer metabolism.

Overall, γδ T cells demonstrate remarkable diversity to small molecules and protein ligands, and are not constrained by antigen-presenting molecules or MHC class I-like proteins. This grants γδ T cells enhanced flexibility in recognizing a variety of tumor-derived and stress-induced signals. γδ T cells, like MAIT cells, often retain cytotoxic potential in immunosuppressive environments and can exhibit partial resistance to exhaustion when phenotypically expressing PD-1^+^CTLA-4^+^TIGIT^+^, making them promising candidates for cancer immunotherapy. However, the duality of their roles, particularly the context-dependent effects of IL-17-producing γδ subsets, warrants careful consideration in therapeutic applications. Ongoing research is needed to elucidate the precise factors governing γδ T cell polarization and function within tumors, including their interactions with other immune cells, metabolic conditions, and cytokine milieu.

## Therapeutic potential of unconventional T cells in cancer

Invariant T cell-based immune therapies are expected to overcome the restriction of the extensive polymorphism of HLA genes in individual patients and the dependence on HLA haplotype-matching for prolonging antigen stimulation. These restricting elements make conventional T cell-based immune therapies costly, time-consuming, and sometimes harmful or toxic in immunopathology. Inaccurate or partial HLA crossmatching can diminish efficacy or lead to adverse events, including graft-versus-host disease. These limitations are evident in recent therapeutic advances involving conventional T cells. For instance, Lifileucel, a tumor-infiltrating lymphocyte (TIL)-based therapy, received FDA approval for treatment-resistant metastatic melanoma after showing durable responses in roughly one-third of patients (164). Another example came with afamitresgene autoleucel, a TCR-engineered T cell therapy developed for HLA-A*02:01-positive synovial sarcoma, which achieved a 40% response rate (165). However, the treatment’s efficacy was constrained by its reliance on HLA restriction, confining eligibility to patients expressing HLA-A2 subtypes and still posing a risk of ‘on-target, off-tumor effects’ toxicities, where healthy tissues are inadvertently targeted, leading to severe or fatal side effects (166).

In this context, unconventional T cells offer an exciting alternative platform for extending the benefits of T cell-based immunotherapies to a wider patient base. Invariant T cells express conserved TCR domains that enable population-wide responses with minimal variability. Unlike the highly diverse αβ TCR repertoire of conventional T cells, which exhibits vast signatures up to 10^11^ variations within a single individual (167), invariant TCRs confer broad antigen recognition with limited specificity, allowing a rapid and innate-like activation. It is expected that responses in early tumor surveillance will lead to early detection of malignancies and early defense against emerging malignant cells by the existing high baseline frequency of unconventional T cells in tissues. This rapid responding kinetics of unconventional T cells occurs prior to the prolonged cancer antigen-stimulation and clonal expansion of conventional T cells, which often take place alongside the establishment of TME-mediated suppression and exhaustion under chronic stimulation (168). Unconventional T cells, while not associated with traditional memory, exhibit greater resistance to exhaustion than conventional αβ T cells (169), retain cytotoxic potential in certain immune-suppressive environments, bridge innate and adaptive responses, making them valuable in immunotherapy contexts. Their ability to recognize metabolite ligands through semi-invariant TCRs independent of peptide antigen processing and HLA restriction could bypass one of the key bottlenecks in current adoptive T cell approaches. Concepts like TIL therapy and TCR-engineered strategies, already proven in conventional T cells, could be adapted for invariant T cells by exploiting their unique recognition pathways.

### NKT cell therapies

Most importantly, three different invariant T cell populations respond to distinct metabolite compounds. CD1-restricted T cells, mainly CD1d-restricted NKT cells, have been intensively tested in numerous pre-clinical studies (90, 111), as briefly summarized in **Table 4**. These studies generally apply α-GalCer in mouse models, stimulate dendritic cells with α-GalCer, transfer *ex vivo*-expanded NKT cells, expand NKT cells with α-GalCer-loaded CD1d protein, or generate invariant NKT cells with chimeric antigen receptor (CAR) expression, mostly leading to a prolonged survival or inhibited tumor growth in animals bearing metastatic tumor cells (108, 170–173). The α-GalCer-loaded APCs and synthetic glycolipid agonists can expand and activate iNKT cells, skewing them toward Th1-like subsets that favor antitumor responses. Modified α-GalCer analogs and nanoparticle delivery systems may potentiate iNKT-mediated anti-tumor responses (174, 175). Strategies to overcome iNKT cell anergy, such as anti-PD-1 checkpoint blockade and cytokine supplementation with IL-2 or G-CSF, are promising approaches. In one study, a blockade of PD-1/PD-L interactions during α-GalCer treatment preserved iNKT cell responsiveness and enhanced anti-metastatic activity by restoring IFN-γ production and NK cell cytotoxicity (104). Both autologous and allogeneic iNKT cells have shown safety and early signs of efficacy in clinical trials, while monoclonal antibody-based approaches have demonstrated the ability to either expand iNKT cells to enhance antitumor responses or deplete them in settings where they may promote tumor growth (176). To test the anti-cancer efficacy of NKT cells in humans, CAR-NKT cell therapies and recombinant TCRs (rTCRs) are under active investigation in clinical trials, including using direct α-GalCer injection (177), α-GalCer-pulsed monocyte-derived dendritic cells (178), adoptive transfer of autologous *ex vivo*-expanded NKT cells (179), and some trials with combined therapies (180), with overall outcomes of stable diseases or effectiveness in a small percentage of patients. It also appears that combined therapies may increase the efficacy of therapy in patients with head and neck squamous cell carcinoma using respective injection of expanded NKT cells and α-GalCer-pulsed blood cells to observe objective tumor regression in 50% of patients (180, 181). Expanding IFNγ-producing NKT cells in tumor tissues likely contributes to an enhanced protection of NKT cell-based anti-cancer immune therapies in multiple clinical trials.

### γδ T cell therapies

Through responding to various stress-induced metabolite or protein ligands, an adoptive transfer of activated γδ T cells is promising as a candidate for immunotherapy. These innate-like T cells have shown greater resistance to exhaustion compared to αβ T cells and retained cytotoxicity in checkpoint-inhibited environments. These findings may indicate γδ T cells are ideal for adoptive T cell therapies, particularly in tumors resistant to conventional strategies (182). Approaches proposed or under investigation include the expansion of γδ T cells using phosphoantigen stimulation, followed by reinfusion in combination with cytokines such as IL-2 or IL-15 to enhance persistence and proliferation. Allogeneic γδ T cell therapies are also being discussed to create “off-the-shelf” products, circumventing the need for patient-specific cell manufacturing (183–185). CAR-γδ T cells are in development to enhance tumor specificity, and bispecific antibody platforms are being designed to simultaneously activate both γδ T cells and NK cells. These approaches show particular promise in hematologic malignancies but may also extend to solid tumors with improved cytokine engineering and tumor-homing strategies.

### MR1T cell therapies

Through the activation of polar metabolites from bacterial and mammalian cells, MR1T, including MAIT and diverse MR1T cells, could potentially serve as the foundation for novel cancer vaccines or cell therapies using tumor-derived or synthetic MR1 ligands to drive antitumor responses. Although preclinical or clinical studies remain missing, targeting the MR1-TCR axis with synthetic MR1 ligands is expected to effectively enhance MR1T-mediated cytotoxicity or suppress MR1T cell function when dysregulation contributes to tumor growth. Combination strategies involving PD-1/PD-L1 checkpoint blockade have also been proposed to restore MR1T function in tumors where exhaustion markers are highly expressed, while cytokine support with IL-12 or IL-18 may synergistically boost effector activity (186).

## Current challenges and future perspectives

One conceptual challenge in studying anti-cancer unconventional T cells is a poor understanding of the antigen structures, antigenic specificity, and TCR of unconventional T cells that induce efficient cancer cell killing *in vitro* and inhibit cancer growth *in vivo*. Due to non-peptidic nature of metabolite antigens, robust high-throughput methods are yet missing to identify and validate endogenous tumor-associated ligands presented by MR1 and CD1 via profiling a pool of differential metabolites in cancer vs. normal cells. Although some candidate ligands (e.g., 5-OP-RU or tumor-elevated phospholipids) have been proposed to induce antitumor reactivity, the spectrum of ligands driving optimal antitumor or regulatory responses remains poorly defined. As precise cancer metabolic conditions can shape cancer antigen landscape and specify unconventional T cell responses, knowledge is currently missing to link metabolic pathways and products in cancer cells with unconventional T cell activation, warranting further investigation of cancer cell-derived metabolite antigens.

A further conceptual challenge is to define tumor-specific and tumor-associated antigens, which have been used to differentiate peptide antigens that are solely expressed or just enriched in cancer cells. For polar metabolite antigens, little is known about their expression in cancer versus normal cells, because the definition of tumor-specific or associated metabolite antigens has to be validated by mass spectrometry or NMR detection of metabolite compounds in cells or from MHC class I-like proteins between various cancer cells and normal cells, comparatively. For the newly reported nucleoside or nucleobase analogs from a preprint or peer-reviewed articles for MAIT cell or diverse MR1T activation (18–20), their expression levels in cancer vs. normal cells remain unknown. For lipid metabolites that activate iNKT cells, determining the expression levels of tumor-associated CD1-presented lipid antigens is equally crucial. Thus, a full range of tumor-associated or specific metabolite antigens and their *in vivo* relevance remain elusive. Further, whether effector MAIT cells or NKT cells rely on differential expression levels of metabolite antigens to differentiate cancer cells from normal cells is also an imperative question in future studies.

Technical challenges also hamper the generation and delivery of unconventional T cell-targeted reagents for therapies. Major efforts are needed to cultivate and engineer unconventional T cells for expressing cancer tissue-targeting molecules, such as CAR-MAIT targeting the Her2 protein in breast cancer, CD19-targeted CAR-T against B cell malignancy, or tissue-homing chemotaxis receptors, allowing unconventional T cells to better reach cancer tissues. Although preclinical models show promise (**Table 4**), it remains unclear from a clinical standpoint whether the adoptively transferred unconventional T cells can efficiently migrate to and be detected in cancer tissues for cancer cell killing. Technological barriers to clinical translation also include the need for improved ligand delivery systems that enable stable, tumor-specific MR1 or CD1 ligand loading *in vivo*. Metabolite antigens are different from peptide antigens in chemical nature, therefore, the chemical formulations designed for delivering peptide antigens are likely not optimal for delivering polar and lipid metabolites. Early studies for delivering α-GalCer and other glycolipids, such as PEGylated (polyethylene glycol-formulated), lipid nanocarriers (187), liposome formulations with glycolipids or peptides (188–190), and codelivery with mRNA (191), are valuable to be further tested in lipid and polar metabolite antigen delivery.

Challenges in translational applications are also multifold. Long-term therapeutic efficacy usually depends on maintaining effector memory phenotypes and minimizing T cell exhaustion within the immunosuppressive tumor microenvironment (TME), involving strategies to enhance T cell infiltration and retention within solid tumors. This may require the development of metabolic reprogramming approaches, cytokine support systems (e.g., IL-7 or IL-15 co-administration), or engineering of resistance to TME-associated inhibitory pathways. Thus, a central challenge for the clinical success of unconventional T cell immunotherapies is to understand how MR1- or CD1-restricted T cells can be sustainably maintained with durable responses, while resisting cancer-driven immunosuppression and T cell exhaustion within tumor environments. Similar to the importance of correct peptide antigens in inducing sustainable conventional T cell responses for protection, the identification of metabolite antigens to induce durable unconventional T cell protection against cancer is a feasible method for designing novel anti-cancer therapies. However, different from the protective peptide antigens specifically restricted by individual HLA alleles, a protective metabolite antigen, if identified, can be applied to all patients as a universal treatment, regardless of their diverse HLA genetics.

A further relevant critical gap is the lack of clinically validated methods to monitor unconventional T cell trafficking and activity in real time. Emerging technologies such as non-invasive imaging using labeled TCR ligands, single-cell transcriptomics of tumor-infiltrating lymphocytes, and circulating T cell receptor repertoire profiling, and antigen loading on antigen-presenting cells for antitumor T cell activation offer promising avenues to track dynamic T cell response during therapy. Furthermore, the development of preclinical models that accurately recapitulate invariant T cell function in human tumors is needed to accelerate clinical translation. Humanized mouse models considering their comparative differences (192) offer promising platforms to evaluate the therapeutic potential of unconventional T cells in diverse cancer settings.

Together, a better understanding of antigen presentation dynamics, TCR repertoire diversity, and intratumoral localization of these T cells is needed to inform therapeutic design. Ultimately, bridging ligand discovery, engineering strategies for T cell persistence, and real-time monitoring tools will be key to unlocking the full clinical potential of these unconventional T cell populations in cancer immunotherapy (136). These efforts underscore the expanding frontier of T cell-based immunotherapies, where invariant T cell subsets may soon complement or enhance the current landscape shaped by conventional T cells.
